# Supplementary material for: Mapping the immunogenic landscape of near-native HIV-1 envelope trimers in non-human primates
Source: PLoS Pathog. 2020 Aug 31;16(8):e1008753. doi: 10.1371/journal.ppat.1008753 (PMC7485981; doi:10.1371/journal.ppat.1008753)
Supplement: S1 Fig — (A) FACS gating strategy for isolation of BG505 SOSIP specific memory B-cells. (B) BG505 SOSIP.664 trimer and BG505 gp120 ELISA binding data for mAbs isolated from RM rh1987 and (C) from RM rh2011. (PDF) [file ppat.1008753.s001.pdf]

A

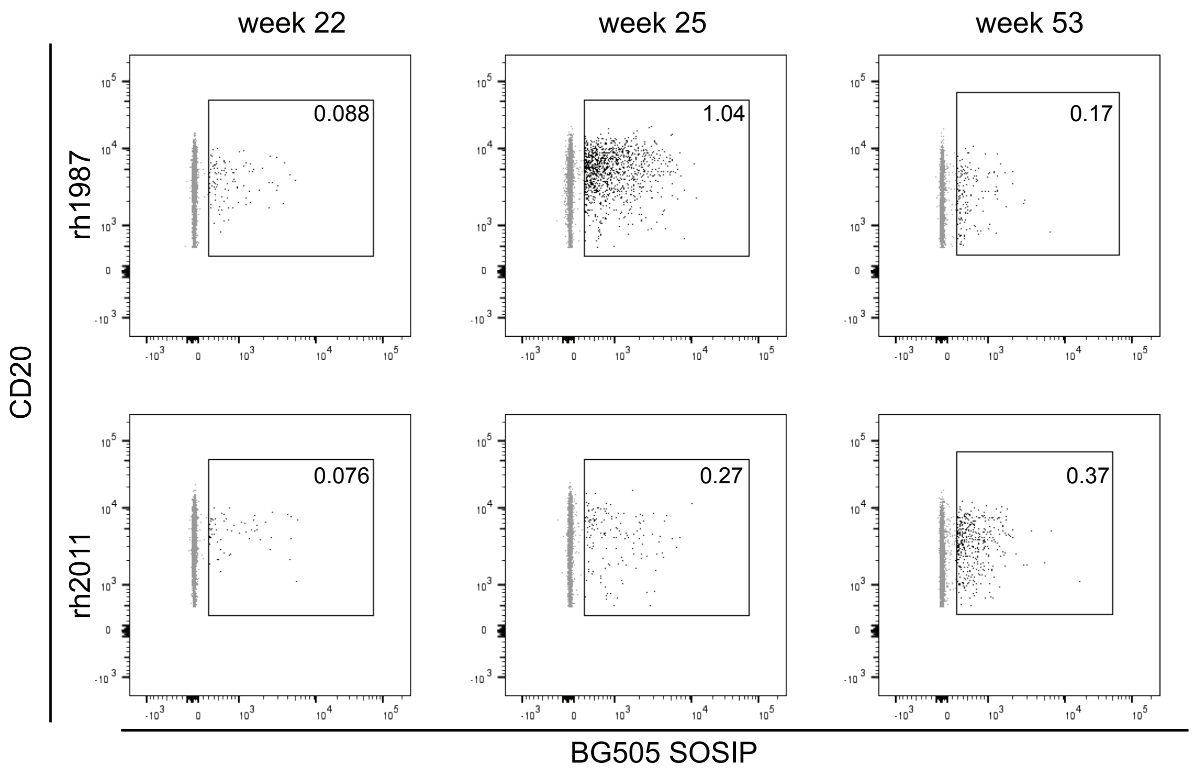

B

mAb	SOSIP ELISA	gp120 ELISA
RM19A	++	+++
RM19A1	+++	+++
RM19A2	+++	+++
RM19A3	++	+
RM19B	++	-
RM19B1	+	-
RM19C	+	-
RM19C2	++	-
RM19C3	++	-
RM19C4	++	+
RM19D	+++	+++
RM19E	+	-
RM19F	+	-
RM19F1	++	-
RM19G	++	-
RM19J	++	-
RM19K	+++	++
RM19L	++	-
RM19M	++	-
RM19N	+	+
RM19O	+++	+++
RM19P	+++	+++
RM19R	++	-
RM19S	++	-
RM19T	++	++

C

mAb	SOSIP ELISA	gp120 ELISA
RM20A	++	-
RM20A1	++	-
RM20A2	++	-
RM20A3	++	-
RM20B	+	-
RM20B1	+	-
RM20C	+	-
RM20D	++	+++
RM20E	+++	+
RM20E1	++	+
RM20E2	++	+
RM20E3	+	+
RM20F	++	+
RM20G	++	-
RM20H	++	++
RM20I	++	++
RM20J	+++	+++

EC50 (ug/mL)	
<0.1	+++
0.1-1	++
1-10	+
>10	-

S1 Fig. MAb isolation and characterization from BG505 SOSIP.664 trimer-immunized macaques.
(A) FACS gating strategy for isolation of BG505 SOSIP specific memory B-cells. (B) BG505 SOSIP.664 trimer and BG505 gp120 ELISA binding data for mAbs isolated from RM rh1987 and (C) from RM rh2011.
