# Supplementary material for: Mapping the immunogenic landscape of near-native HIV-1 envelope trimers in non-human primates
Source: PLoS Pathog. 2020 Aug 31;16(8):e1008753. doi: 10.1371/journal.ppat.1008753 (PMC7485981; doi:10.1371/journal.ppat.1008753)
Supplement: S2 Fig — Representative 2D class averages, 3D reconstructions, and EMDB accession numbers. (PDF) [file ppat.1008753.s002.pdf]

A

mAb	2D class averages	3D reconstructions	Epitope	EMDB#
RM19A1	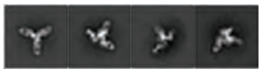	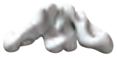	289 GH	EMD-21062
RM19B	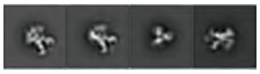	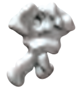	base	EMD-21075
RM19B1	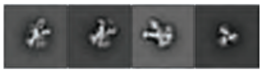	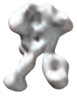	base	EMD-21077
RM19C	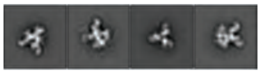	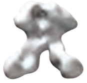	base	EMD-21078
RM19C2	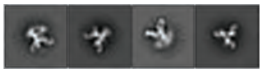	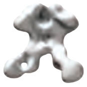	base	EMD-21079
RM19C3	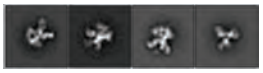	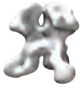	base	EMD-21082
RM19E	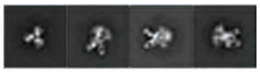	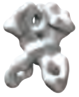	base	EMD-21080
RM19F	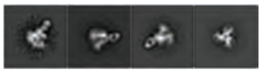	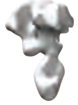	base	EMD-21056
RM19G	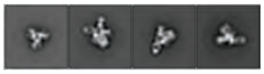	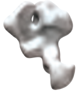	base	EMD-21081
RM19J	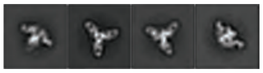	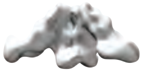	289 GH	EMD-21055
RM19K	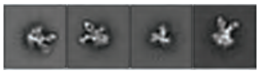	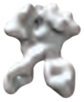	base	EMD-21076
RM19L	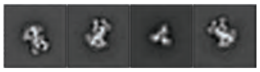	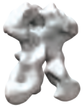	base	EMD-21066
RM19M	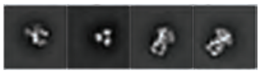	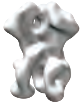	base	EMD-21061
RM19N	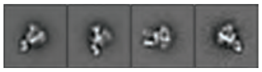	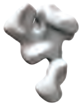	base	EMD-21053
RM19O	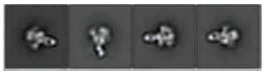	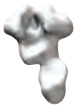	base	EMD-21065
RM19P	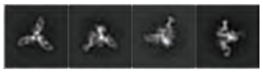	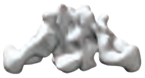	289 GH	EMD-21064
RM19R	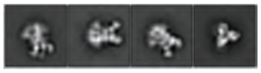	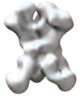	base	EMD-21058
RM19S	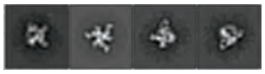	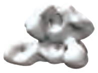	N611/FP	EMD-21059
RM19T	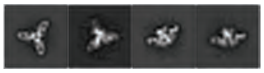	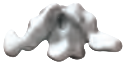	N289 GH	EMD-21057

B

mAb	2D class averages	3D reconstructions	Epitope	EMDB#
RM20A2	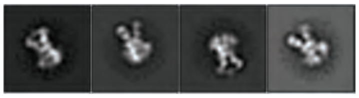	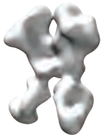	base	EMD-21083
RM20A3	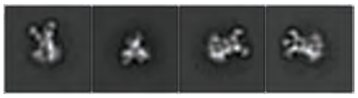	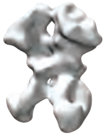	base	EMD-21084
RM20B	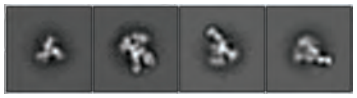	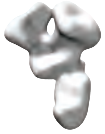	base	EMD-21085
RM20B1	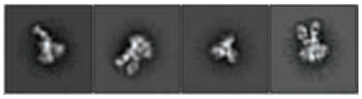	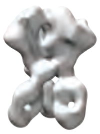	base	EMD-21086
RM20C	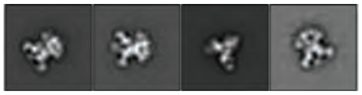	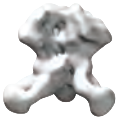	base	EMD-21087
RM20E	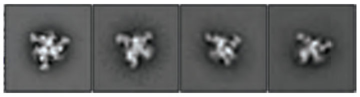	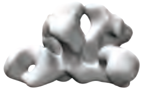	N611/FP	EMD-21093
RM20E1	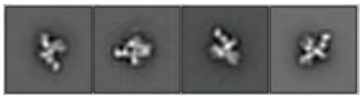	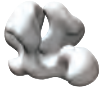	N611/FP	EMD-21090
RM20F	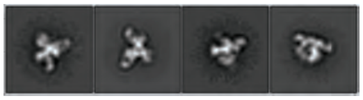	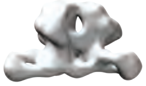	gp120/gp41 interface	EMD-21091
RM20G	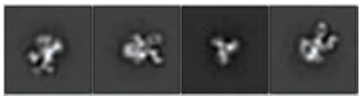	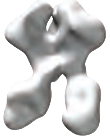	base	EMD-21088
RM20H	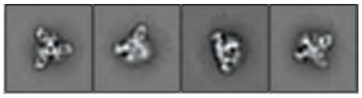	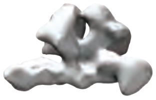	gp120/gp41 interface	EMD-21092
RM20J	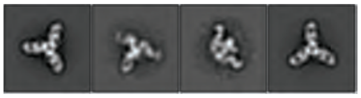	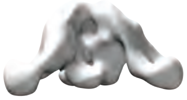	289 GH	EMD-21089

S2 Fig. Negative stain electron microscopy epitope mapping of Fabs from rh1987 and rh2011.
Representative 2D class averages, 3D reconstructions, and EMDB accession numbers for (A) rh1987 and (B) rh2011.
