# Supplementary material for: Mapping the immunogenic landscape of near-native HIV-1 envelope trimers in non-human primates
Source: PLoS Pathog. 2020 Aug 31;16(8):e1008753. doi: 10.1371/journal.ppat.1008753 (PMC7485981; doi:10.1371/journal.ppat.1008753)
Supplement: S3 Fig — (A) Representative micrographs for cryoEM datasets. (B) Local resolution maps for each complex generated in cryoSPARC v2 (Punjani et al., 2017). (C) Gold-standard Fourier shell correlation (FSC) curves for each complex showing global resolution calculated at FSC = 0.143. (D) Crystal structures of unliganded Fabs RM20J, RM20F, and RM20E1. Heavy chains are shown in green and light chains shown in light blue. (PDF) [file ppat.1008753.s003.pdf]

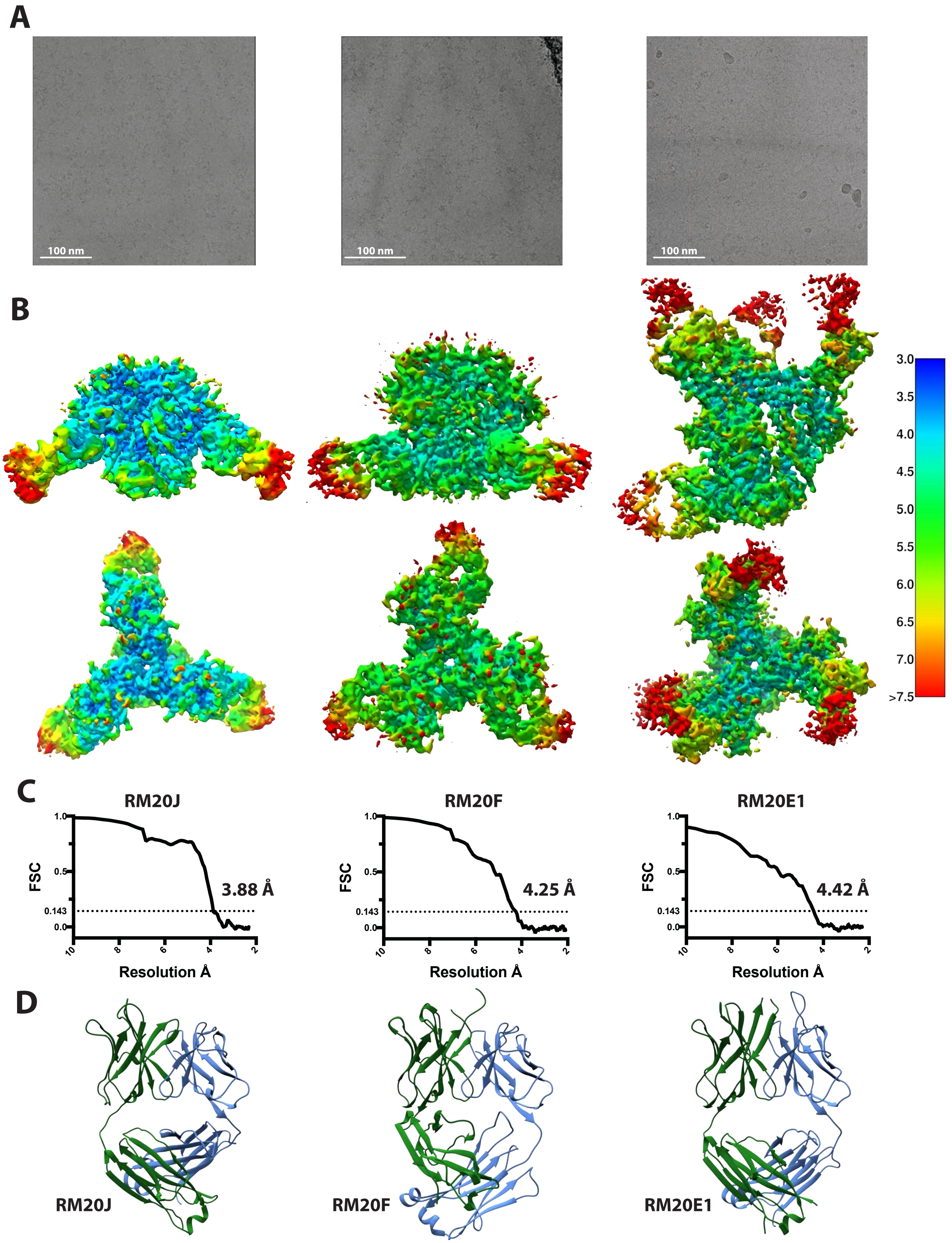

S3 Fig. Cryo-EM structures of BG505 SOSIP trimers with mAbs and crystal structures of mAbs. (A) Representative micrographs for cryoEM datasets. (B) Local resolution maps for each complex generated in cryoSPARC v2 (Punjani et al., 2017). (C) Gold-standard Fourier shell correlation (FSC) curves for each complex showing global resolution calculated at FSC = 0.143. (D) Crystal structures of unliganded Fabs RM20J, RM20F, and RM20E1. Heavy chains are shown in green and light chains shown in light blue.
