# Supplementary material for: Mapping the immunogenic landscape of near-native HIV-1 envelope trimers in non-human primates
Source: PLoS Pathog. 2020 Aug 31;16(8):e1008753. doi: 10.1371/journal.ppat.1008753 (PMC7485981; doi:10.1371/journal.ppat.1008753)
Supplement: S4 Fig — The HIV Env trimer is shown as a surface representation with one protomer colored in grey, the other two protomers colored in blue, and the N-linked glycans colored in green. The FP (residues 512–522) from the antibody bound structures of RM20E1, RM20F, DFPH-a.15 (PDB: 6N1W), VRC34 (PDB: 5I8H), and ACS202 (PDB: 6NC2) are shown as colored backbone ribbon diagrams. (PDF) [file ppat.1008753.s004.pdf]

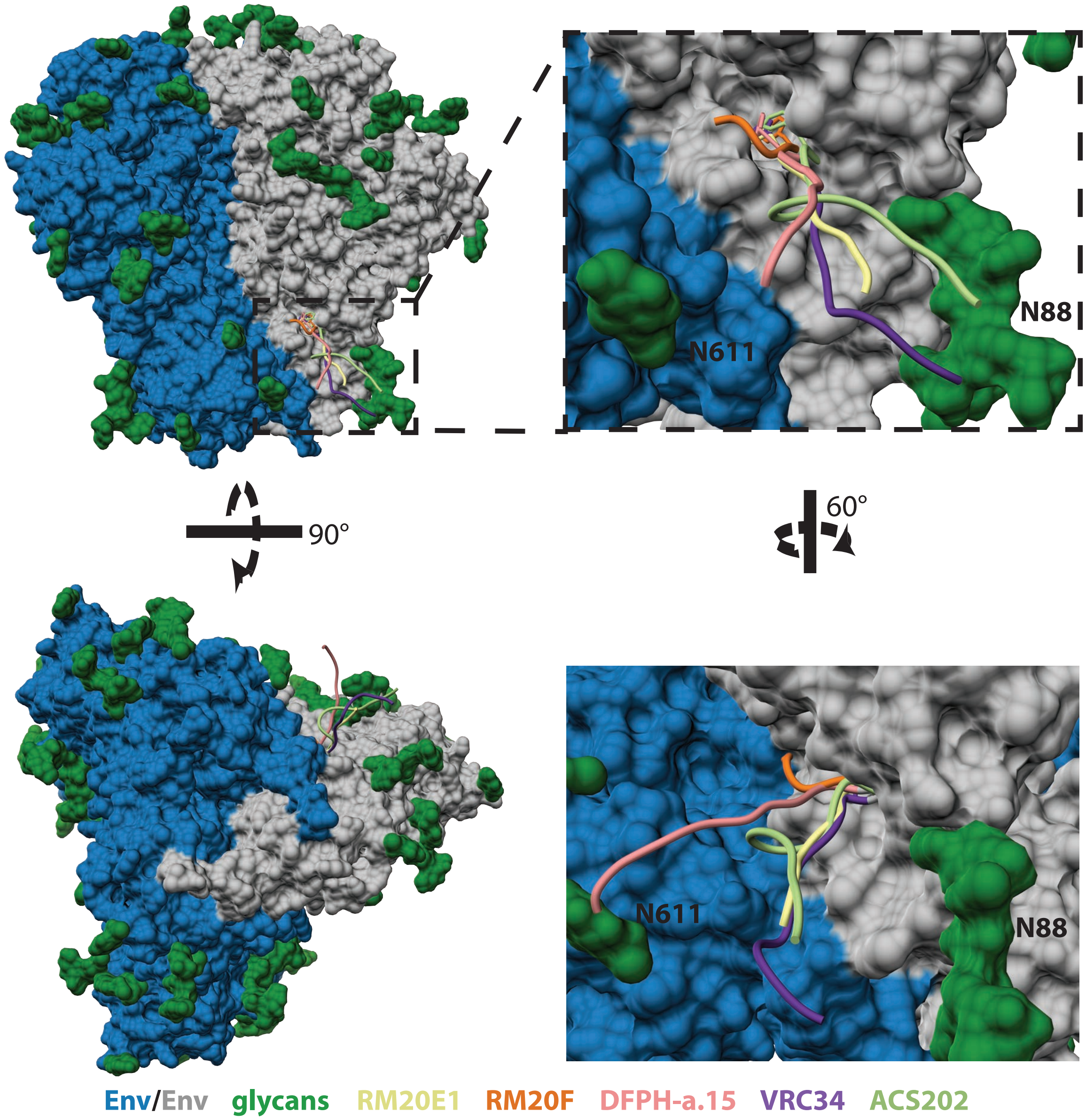

S4 Fig. Antibody bound FP conformations. The HIV Env trimer is shown as a surface representation with one protomer colored in grey, the other two protomers colored in blue, and the N-linked glycans colored in green. The FP (residues 512-522) from the antibody bound structures of RM20E1, RM20F, DFPH-a.15 (PDB: 6N1W), VRC34 (PDB: 5I8H), and ACS202 (PDB: 6NC2) are shown as colored backbone ribbon diagrams.
