# Supplementary material for: Mapping the immunogenic landscape of near-native HIV-1 envelope trimers in non-human primates
Source: PLoS Pathog. 2020 Aug 31;16(8):e1008753. doi: 10.1371/journal.ppat.1008753 (PMC7485981; doi:10.1371/journal.ppat.1008753)
Supplement: S2 Table — (PDF) [file ppat.1008753.s006.pdf]

S2 Table. MAb Characteristics

mAb	Timepoint	Animal ID	LC type	VH gene	DH gene	JH gene	HCDR3 aa	HCDR3 length	HC %SHM	VL gene	JL gene	LCDR3 aa	LCDR3 length	LC %SHM	Epitope targeted
RM19A	week 22	rh1987	Kappa	IGHV3-AFE*01_S3736	IGHD4-22*01	IGHJ4*01	ARGGVYSNYEF	11	4.6%	IGKV1-AAL*01	LJI.Rh_IGKJ1.1	QQYNSYPPT	9	5.6%	289 hole epitope*
RM19A1	week 25	rh1987	Kappa	IGHV3-AFE*01_S3736	IGHD4-22*01	IGHJ4*01	AKGGVYSNYDF	11	6.3%	IGKV1-AAL*01	LJI.Rh_IGKJ1.1	QQYNSDPPT	9	4.7%	289 hole epitope
RM19A2	week 25	rh1987	Kappa	IGHV3-AFE*01_S3736	IGHD4-22*01	IGHJ4*01	AKGGVYSNYDF	11	7.2%	IGKV1-AAL*01	LJI.Rh_IGKJ1.1	QQYNSDPPT	9	5.9%	289 hole epitope*
RM19A3	week 25	rh1987	Kappa	IGHV3-AFE*01_S3736	IGHD4-22*01	IGHJ4*01	ARGGVYSNYDY	11	3.4%	IGKV1-AAL*01	LJI.Rh_IGKJ1.1	QQHNRDPPT	9	4.3%	289 hole epitope*
RM19D	week 22	rh1987	Kappa	LJI.Rh_IGHV3.50	IGHD3-26*01	IGHJ6*01	VRDYYGGGLD	10	3.3%	IGKV2-AEB*01	LJI.Rh_IGKJ2.2	TQGLEFPYS	9	2.6%	n.d.
RM19J	week 25	rh1987	Kappa	IGHV4-AGU*01_S2780	IGHD4-I1	IGHJ4*01	ARFNSGDYDF	10	8.6%	IGKV1-AAL*01	LJI.Rh_IGKJ3.3	INIILTLSL	9	8.7%	289 hole epitope
RM19N	week 25	rh1987	Kappa	IGHV4-S40_S2532	IGHD2-8*01	IGHJ4*01	ARERVVSATSDVYYFDY	17	2.1%	IGKV1-S57	LJI.Rh_IGKJ2.2	QHSYDTPYS	9	4.4%	base
RM19P	week 25	rh1987	Kappa	IGHV4-AGU*01_S2780	IGHD6-29*01	IGHJ4*01	SSSGWSV	7	4.6%	IGKV1-AAY*01	LJI.Rh_IGKJ2.2	QQYNSYPYS	9	3.7%	289 hole epitope
RM19R	week 25	rh1987	Kappa	IGHV4-S40_S2532	IGHD3-3*01	IGHJ5-1*01	AFFWSTYYKRFDV	13	8.8%	IGKV3-AAP*01_S6980	LJI.Rh_IGKJ1.1	HQENDWPWT	9	5.9%	base
RM19S	week 53	rh1987	Kappa	IGHV4-ABB*01_S8200	IGHD3-9*01	IGHJ4*01	ARRSRRYGDDYGYWYSRSFYFDS	23	7.4%	LJI.Rh_IGKV1.56	LJI.Rh_IGKJ4.4	QQDYSTPFT	9	5.3%	N611/FP
RM19T	week 25	rh1987	Kappa	IGHV4-AGU*01_S2780	IGHD4-I1	IGHJ4*01	ARFNSDYDF	9	9.5%	IGKV1-AAL*01	LJI.Rh_IGKJ3.3	HQHNRYPHT	9	4.7%	289 hole epitope
RM19B	week 22	rh1987	Lambda	IGHV1-AAU*01_S5608	IGHD6-11*01	IGHJ5-2*01	ARIPWRSAIAPGMRGNSFDV	20	4.4%	IGLV2-ABJ*01_S9052	IGLJ1-S1	SSYAGSNSYI	10	4.3%	base
RM19B1	week 22	rh1987	Lambda	IGHV1-AAU*01_S5608	IGHD1-I7	IGHJ5-2*01	ARIMWRSAIAPGMRGNSMDV	20	6.7%	IGLV2-ABJ*01_S9052	IGLJ1-S1	SSYGGSNTYI	10	4.6%	base
RM19C	week 22	rh1987	Lambda	IGHV4-AGU*01_S6650	IGHD2-8*01	IGHJ5-2*01	ARYCRGSICYGGGSLDV	17	7.9%	LJI.Rh_IGLV2.21	IGLJ2-S2	SSYAGSNTLV	10	1.6%	base
RM19C2	week 25	rh1987	Lambda	IGHV4-AGU*01_S6650	IGHD2-30*01	IGHJ5-2*01	ARYCSGSLCYGGGSLDV	17	9.7%	LJI.Rh_IGLV2.21	IGLJ2-S2	SSYAGSNTLV	10	3.1%	base
RM19C3	week 25	rh1987	Lambda	IGHV4-AGU*01_S6650	IGHD2-8*01	IGHJ5-2*01	ARYCSGALCYGGGSLDV	17	7.3%	LJI.Rh_IGLV2.21	IGLJ3-S3	SSYAGSNTLL	10	1.5%	base
RM19C4	week 25	rh1987	Lambda	IGHV4-AGU*01_S6650	IGHD2-2*01	IGHJ5-2*01	ARYCRGSICYGGGSLDV	17	10.2%	LJI.Rh_IGLV2.21	IGLJ3-S3	SSYAGNNILF	10	2.5%	base*
RM19E	week 22	rh1987	Lambda	LJI.Rh_IGHV3.122_S8388	IGHD2-35*01	LJI.Rh_IGHJ5.4	VRVVVSATRGDHFDV	15	6.3%	LJI.Rh_IGLV8.111	IGLJ6-S6	LIYMGSGISV	10	5.9%	base
RM19F	week 22	rh1987	Lambda	LJI.Rh_IGHV4.79.a_S9501	IGHD2-35*01	IGHJ4*01	ARFLVVSAIKAATVPLEY	18	3.9%	IGLV2-ABE*01	IGLJ3-S3	SSYAGSNTYVL	11	0.6%	base
RM19F1	week 22	rh1987	Lambda	LJI.Rh_IGHV4.79.a	IGHD2-35*01	IGHJ4*01	ARFLVVSAIKAATVPLEY	18	6.2%	IGLV2-ABE*01	IGLJ3-S3	SSYAGSNTYVL	11	0.6%	base*
RM19G	week 22	rh1987	Lambda	IGHV1-AAU*02_S1575	IGHD6-6*01	IGHJ4*01	ARDRLAAAGTEHFDY	15	4.2%	LJI.Rh_IGLV2.33.a	IGLJ3*01	CSYLSGSTWV	10	3.1%	base
RM19K	week 25	rh1987	Lambda	IGHV1-AAU*01_S5608	IGHD3-26*01	IGHJ5-2*01	ARGLDYYGARQLGNSLHV	18	6.1%	LJI.Rh_IGLV2.29	IGLJ3*01	YSYAGSNTWV	10	2.8%	base
RM19L	week 25	rh1987	Lambda	LJI.Rh_IGHV4.67.a	IGHD2-35*01	IGHJ3*01	ARAPSYGGTSWAFSEGFDF	19	6.2%	IGLV11-S42	IGLJ2-S2	QMYGSSGAL	9	3.6%	base
RM19M	week 25	rh1987	Lambda	IGHV4-AGU*01_S2780	IGHD3-9*01	LJI.Rh_IGHJ5.4	ARDGYENDYGFYHPVRHRFDV	21	6.6%	IGLV2-ABU*02	IGLJ3*01	FSYTTNKTWV	10	10.7%	base
RM19O	week 25	rh1987	Lambda	IGHV4-Korf3	IGHD3-26*01	IGHJ4*01	AAGGGYYGNHFDF	13	5.9%	LJI.Rh_IGLV2.21_S2946	IGLJ1*01	SSYAGSSTHLF	11	2.8%	base
RM20E	week 53	rh2011	Kappa	IGHV5-ABI*01_S2502	IGHD1-7*01	IGHJ5-1*01	VMWVYILTTGNIWVDV	16	6.4%	LJI.Rh_IGKV2.71	LJI.Rh_IGKJ2.2	GQITDFPYS	9	3.6%	N611/FP
RM20E1	week 53	rh2011	Kappa	IGHV5-ABI*01_S2502	IGHD1-7*01	IGHJ5-1*01	VMWVYILTTGNIWVDV	16	5.4%	LJI.Rh_IGKV2.71	LJI.Rh_IGKJ2.2	GQITDFPYS	9	3.6%	N611/FP
RM20E2	week 53	rh2011	Kappa	IGHV5-ABI*01_S2502	IGHD1-7*01	IGHJ5-1*01	VMWVYILTTGNIWVDV	16	5.1%	LJI.Rh_IGKV2.71	LJI.Rh_IGKJ2.2	GQITDFPYS	9	4.5%	N611/FP*
RM20E3	week 53	rh2011	Kappa	IGHV5-ABI*01_S2502	IGHD1-7*01	IGHJ5-1*01	VMWVYILTTGNIWVDV	16	5.4%	LJI.Rh_IGKV2.71	LJI.Rh_IGKJ2.2	GQGTDFPYS	9	3.0%	N611/FP*
RM20F	week 53	rh2011	Kappa	IGHV3-AFY*10	IGHD3-14*01	IGHJ2-I1	ARGGKPIYYSGGYPSWYFDL	20	7.6%	IGKV6-I1	LJI.Rh_IGKJ3.3	QQTNSFPCT	9	5.3%	gp120/gp41 interface
RM20H	week 53	rh2011	Kappa	LJI.Rh_IGHV3.76.a_S4190	IGHD2-13*01	IGHJ5-1*01	AKDWVGDYYTGTYPGFWFDV	20	5.9%	IGKV3-ADW*01	LJI.Rh_IGKJ4.4	QQNSNWPLT	9	4.0%	gp120/gp41 interface
RM20I	week 53	rh2011	Kappa	IGHV4-ACQ*01_S4946	IGHD3-3*01	IGHJ4*01	ARRVSRLDFGGAFDC	15	6.2%	LJI.Rh_IGKV2.71.a	LJI.Rh_IGKJ4.4	GQGTHWPLT	9	4.8%	n.d.
RM20J	week 53	rh2011	Kappa	IGHV4-AGU*01_S6650	IGHD1-1*01	IGHJ4*01	ARWSTADFDY	10	8.2%	IGKV1-AAL*01	LJI.Rh_IGKJ3.3	QQHNNYPLT	9	5.3%	289 hole epitope
RM20A	week 22	rh2011	Lambda	IGHV3-AFE*01_S3736	IGHD6-29*01	IGHJ4*01	AKGGMSSAWQSSKYYFDF	18	5.6%	LJI.Rh_IGLV2.21_S2946	IGLJ1-S1	SSYAGSKTFYI	11	4.0%	base*
RM20A1	week 22	rh2011	Lambda	IGHV3-AFE*01_S3736	IGHD6-29*01	IGHJ4*01	ARGGMSSAWQSSKYYFDQ	18	8.5%	LJI.Rh_IGLV2.21_S2946	IGLJ1*01	SSYAGRNTFYV	11	5.3%	base*
RM20A2	week 25	rh2011	Lambda	IGHV3-AFE*01_S3736	IGHD6-29*01	IGHJ4*01	ARGGMSSAWQSSKYYFDQ	18	9.1%	LJI.Rh_IGLV2.21_S2946	IGLJ1*01	SSYAGRNTFYV	11	4.7%	base
RM20A3	week 53	rh2011	Lambda	IGHV3-AFE*01_S3736	IGHD6-29*01	IGHJ4*01	ATGGMSSALQSSKYYFDF	18	9.1%	LJI.Rh_IGLV2.21_S2946	IGLJ1-S1	SAYAGRQTFYI	11	4.6%	base
RM20B	week 25	rh2011	Lambda	IGHV4-S11	IGHD2-35*01	IGHJ5-1*01	ARLLVSAIRWEDRFDV	16	3.0%	LJI.Rh_IGLV2.18	IGLJ1-S1	SSLGGSGTYI	10	5.0%	base
RM20B1	week 25	rh2011	Lambda	IGHV4-S11_S0312	IGHD2-35*01	IGHJ5-1*01	ARLLVSAIRWEDRFDV	16	3.8%	LJI.Rh_IGLV2.18	IGLJ1*01	SSFAGGGTYI	10	4.5%	base
RM20C	week 25	rh2011	Lambda	IGHV3-AEW*01	IGHD3-9*01	IGHJ4*01	TRVAYEHDYGYYYKGYYFDF	20	4.4%	IGLV1-ACV*01	IGLJ6-S6	QSYDSSLSAHV	11	3.7%	base
RM20D	week 25	rh2011	Lambda	IGHV3-AFY*05	IGHD4-15*01	IGHJ4*01	ARDPSRYGNYPDN	13	3.6%	LJI.Rh_IGLV4.105	IGLJ3-S3	QTWTAGIVS	9	1.8%	n.d.
RM20G	week 53	rh2011	Lambda	IGHV3-AEW*01	LJI.Rh_IGHD5.29	IGHJ6*01	TRMRFASAQEPVYYGMD	17	6.6%	IGLV1-S16_S2133	IGLJ1-S1	AAWDDSLGGYI	11	3.4%	base

*Inferred based on clonal relationship to mAbs with epitopes mapped by ns-EM.
Clonal families are highlighted with individual colors.
