# Supplementary material for: Mapping the immunogenic landscape of near-native HIV-1 envelope trimers in non-human primates
Source: PLoS Pathog. 2020 Aug 31;16(8):e1008753. doi: 10.1371/journal.ppat.1008753 (PMC7485981; doi:10.1371/journal.ppat.1008753)
Supplement: S3 Table — (PDF) [file ppat.1008753.s007.pdf]

S3 Table. MAb neutralization, ELISA binding, and EM epitope mapping.

		Neutralization IC_{50} (µg/mL)		Binding EC_{50}		
**mAb**	**Timepoint**	**BG505 WT**	**BG505 N611A**	**SOSIP ELISA**	**gp120 ELISA**	**Epitope targeted**
RM19A	week 22	41.8	>50	++	+++	289 hole epitope*
RM19A1	week 25	44.9	>50	+++	+++	289 hole epitope
RM19A2	week 25	>50	>50	+++	+++	289 hole epitope*
RM19A3	week 25	>50	<0.41	++	+	289 hole epitope*
RM19B	week 22	n.d.	n.d.	++	-	base
RM19B1	week 22	>50	>50	+	-	base
RM19C	week 22	n.d.	n.d.	+	-	base
RM19C2	week 25	n.d.	n.d.	++	-	base
RM19C3	week 25	>50	>50	++	-	base
RM19C4	week 25	>50	>50	++	+	base*
RM19D	week 22	>50	>50	+++	+++	n.d.
RM19E	week 22	n.d.	n.d.	+	-	base
RM19F	week 22	n.d.	n.d.	+	-	base
RM19F1	week 22	n.d.	n.d.	++	-	base*
RM19G	week 22	n.d.	n.d.	++	-	base
RM19J	week 25	>50	>50	++	-	289 hole epitope
RM19K	week 25	n.d.	n.d.	+++	++	base
RM19L	week 25	n.d.	n.d.	++	-	base
RM19M	week 25	12.9	5.63	++	-	base
RM19N	week 25	>50	>50	+	+	base
RM19O	week 25	>50	25.5	+++	+++	base
RM19P	week 25	23.5	>50	+++	+++	289 hole epitope
RM19R	week 25	>50	>50	++	-	base
RM19S	week 53	>50	3.51	++	-	N611/FP
RM19T	week 25	>50	>50	++	++	289 hole epitope
RM20A	week 22	n.d.	n.d.	++	-	base*
RM20A1	week 22	n.d.	n.d.	++	-	base*
RM20A2	week 25	n.d.	n.d.	++	-	base
RM20A3	week 53	>50	>50	++	-	base
RM20B	week 25	>50	>50	+	-	base
RM20B1	week 25	n.d.	n.d.	+	-	base
RM20C	week 25	>50	>50	+	-	base
RM20D	week 25	>50	>50	++	+++	n.d.
RM20E	week 53	>50	<0.41	+++	+	N611/FP
RM20E1	week 53	>50	<0.41	++	+	N611/FP
RM20E2	week 53	>50	<0.41	++	+	N611/FP*
RM20E3	week 53	>50	<0.41	+	+	N611/FP*
RM20F	week 53	1.6	<0.41	++	+	gp120/gp41 interface
RM20G	week 53	n.d.	n.d.	++	-	base
RM20H	week 53	42.9	<0.41	++	++	gp120/gp41 interface
RM20I	week 53	>50	>50	++	++	n.d.
RM20J	week 53	>50	>50	+++	+++	289 hole epitope

*Inferred based on clonal relationship to mAbs with epitopes mapped by ns-EM.

IC_{50} (µg/mL)	EC_{50} (µg/mL)	
<1	<0.1	+++
1-10	0.1-1	++
10-50	1-10	+
>50	>10	-
