# Supplementary material for: Mapping the immunogenic landscape of near-native HIV-1 envelope trimers in non-human primates
Source: PLoS Pathog. 2020 Aug 31;16(8):e1008753. doi: 10.1371/journal.ppat.1008753 (PMC7485981; doi:10.1371/journal.ppat.1008753)
Supplement: S4 Table — (PDF) [file ppat.1008753.s008.pdf]

S4 Table. EM Data Collection and Map/Model Refinement Parameters.

Complex	BG505.v4.1 + RM20F	BG505.v5.2 + RM20J	BG505.v5.2 + RM20E1 + PGT122
Microscope	Titan Krios	Talos Arctica	Talos Arctica
Voltage, kV	300	200	200
Detector	Gatan K2 Sumit	Gatan K2 Sumit	Gatan K2 Sumit
Recording Mode	Counting	Counting	Counting
Magnification	29,000	36,000	36,000
Moive micrograph pixel size, Å	1.03	1.15	1.15
Dose rate, e^-/[(camera pixel)*s]	8.833	6.004	5.656
No. of frames per moive micrograph	24	44	48
Frame exposure time, ms	250	250	250
Movie micrograph exposure time, s	6	11	12
Total dose, $e^-/Å^2$	50.0	49.9	51.3
Defocus range, µm	1.3 to 2.8	1.0 to 3.5	1.0 to 2.5
No. of movie micrographs	1055	553	579
No. of molecular projection images in map	91212	26327	17010
Symmetry	C3	C3	C1
Map resolution (FSC 0.143)	4.25	3.88	4.42
Map sharpening B-factor, $Å^2$	-174.7	-112.6	-81.2
No. of atoms in deposited model	19962	19785	21768
MolProbity score	1.16	1.38	0.97
$C\beta$ Outliers (%)	0.00	0.00	0.00
Rotamer Outliers (%)	0.15	0.72	0.13
Rama Outliers (%)	0.65	1.29	0.20
Clashscore	1.75	2.12	0.97
EMRinger score	2.02	2.71	1.82
Privateer	pass	pass	pass
EMDB	EMD-21246	EMD-21257	EMD-21232
PDB ID	6VN0	6VO1	6VLR
