# Supplementary material for: Mapping the immunogenic landscape of near-native HIV-1 envelope trimers in non-human primates
Source: PLoS Pathog. 2020 Aug 31;16(8):e1008753. doi: 10.1371/journal.ppat.1008753 (PMC7485981; doi:10.1371/journal.ppat.1008753)
Supplement: S5 Table — (PDF) [file ppat.1008753.s009.pdf]

S5 Table. X-ray data collection and refinement statistics.

**Data collection**	**RM20F (PDB:6VSR)**	**RM20J (PDB:6VOS)**	**RM20E1 (PDB:6VOR)**
Beamline	SSRL 12-2	APS 23ID-B	SSRL 12-2
Wavelength (Å)	0.97946	1.03322	0.97946
Space group	$P\ 2_1\ 2_1\ 2_1$	$P\ 4_3\ 2_1\ 2$	P 1
Unit cell parameters	a=61.5, b=75.6, c=115.1, α= β=γ=90	a=b=112.3, c=141.0, α=β=γ=90	a=57.4, b=57.7, c=92.7, α=98.7, β=94.1, γ=97.8
Resolution (Å)	36.7-2.20 (2.24-2.20)[a]	50.0-2.30 (2.34-2.30)[a]	50.0-1.85 (1.90-1.85)[a]
Unique Reflections	28,053 (1,364)[a]	40,057 (1,946)[a]	142,088 (2,413)[a]
Redundancy	4.0 (4.0)[a]	17.5 (11.2)[a]	2.7 (1.3)[a]
Completeness (%)	98.6 (99.4)[a]	100 (99.6)[a]	85.4 (32.5)[a]
$\langle I/\sigma_I \rangle$	7.7 (2.0)[a]	28.0 (1.0)[a]	18.8 (1.2)[a]
R_{sym}[b] (%)	19.7 (83.0)[a]	12.9 (>100)[a]	10.9 (69.1)[a]
R_{pim}[b] (%)	11.0 (46.4)[a]	3.0 (50.3)[a]	4.9 (51.7)[a]
$CC_{1/2}$[c] (%)	83.9 (54.0)[a]	89.7 (44.1)[a]	91.6 (56.0)[a]
	**Refinement statistics**		
Reflections (work)	26,622	39,953	75,965
Reflections (test)	1,374	1,999	3,285
R_{cryst}[d] / R_{free}[e] (%)	19.7/24.1	18.1/21.1	22.4/25.2
No. of atoms			
Protein	3,328	3,604	6,731
Water	288	224	320
Average *B*-value (Å^2)			
Protein	25	62	39
Water	34	68	40
Wilson *B*-value (Å^2)	21	57	30
	**RMSD from ideal geometry**		
Bond length (Å)	0.002	0.007	0.008
Bond angle (°)	0.55	0.95	0.99
	**Ramachandran statistics (%)**		
Favored	97.5	96.1	96.3
Outliers	0.0	0.2	0.1

[a] Numbers in parentheses refer to the highest resolution shell.

[b] $R_{sym} = \Sigma_{hkl} \Sigma_i | I_{hkl,i} - \langle I_{hkl} \rangle | / \Sigma_{hkl} \Sigma_i I_{hkl,i}$ and $R_{pim} = \Sigma_{hkl} (1/(n-1))^{1/2} \Sigma_i | I_{hkl,i} - \langle I_{hkl} \rangle | / \Sigma_{hkl} \Sigma_i I_{hkl,i}$, where $I_{hkl,i}$ is the scaled intensity of the ith measurement of reflection h, k, l, $\langle I_{hkl} \rangle$ is the average intensity for that reflection, and *n* is the redundancy.

[c] $CC_{1/2}$ = Pearson correlation coefficient between two random half datasets.

[d] $R_{cryst} = \Sigma_{hkl} | F_o - F_c | / \Sigma_{hkl} | F_o |$ x 100, where F_o and F_c are the observed and calculated structure factors, respectively.

[e] R_{free} was calculated as for R_{cryst}, but on a test set comprising 5% of the data excluded from refinement.
