# Supplementary material for: Mapping the immunogenic landscape of near-native HIV-1 envelope trimers in non-human primates
Source: PLoS Pathog. 2020 Aug 31;16(8):e1008753. doi: 10.1371/journal.ppat.1008753 (PMC7485981; doi:10.1371/journal.ppat.1008753)
Supplement: S6 Table — (PDF) [file ppat.1008753.s010.pdf]

S6 Table. BLI Binding Kinetics.

mAb	Timepoint	Animal ID	KD (M)	kon(1/Ms)	kdis(1/s)
RM19A	week 22	rh1987	n.d.	n.d.	n.d.
RM19A1	week 25	rh1987	3.94E-10	1.41E+04	5.57E-06
RM19A2	week 25	rh1987	n.d.	n.d.	n.d.
RM19A3	week 25	rh1987	n.d.	n.d.	n.d.
RM19B	week 22	rh1987	9.87E-10	3.12E+05	3.08E-04
RM19B1	week 22	rh1987	7.00E-10	4.01E+05	2.80E-04
RM19C	week 22	rh1987	1.50E-09	2.66E+05	4.00E-04
RM19C2	week 25	rh1987	<1.0E-12	1.12E+05	<1.0E-07
RM19C3	week 25	rh1987	6.80E-11	9.95E+04	6.76E-06
RM19C4	week 25	rh1987	n.d.	n.d.	n.d.
RM19D	week 22	rh1987	1.57E-07	1.68E+03	2.63E-04
RM19E	week 22	rh1987	1.28E-10	2.80E+05	3.60E-05
RM19F	week 22	rh1987	4.98E-09	6.30E+05	3.14E-03
RM19F1	week 22	rh1987	2.88E-08	7.98E+04	2.30E-03
RM19G	week 22	rh1987	3.95E-07	2.67E+03	1.05E-03
RM19J	week 25	rh1987	1.95E-08	3.98E+03	7.77E-05
RM19K	week 25	rh1987	8.55E-08	3.72E+03	3.18E-04
RM19L	week 25	rh1987	6.03E-10	1.02E+05	6.15E-05
RM19M	week 25	rh1987	1.03E-08	2.27E+04	2.34E-04
RM19N	week 25	rh1987	1.46E-08	3.66E+05	5.33E-03
RM19O	week 25	rh1987	1.87E-08	3.82E+04	7.14E-04
RM19P	week 25	rh1987	2.09E-09	8.26E+03	1.73E-05
RM19R	week 25	rh1987	5.52E-10	1.12E+05	6.18E-05
RM19S	week 53	rh1987	1.47E-07	7.16E+03	1.05E-03
RM19T	week 25	rh1987	8.45E-10	2.28E+04	1.93E-05
RM20A	week 22	rh2011	n.d.	n.d.	n.d.
RM20A1	week 22	rh2011	n.d.	n.d.	n.d.
RM20A2	week 25	rh2011	<1.0E-12	8.95E+05	<1.0E-07
RM20A3	week 53	rh2011	<1.0E-12	7.82E+05	<1.0E-07
RM20B	week 25	rh2011	3.92E-08	1.67E+04	6.56E-04
RM20B1	week 25	rh2011	1.85E-09	3.48E+05	6.44E-04
RM20C	week 25	rh2011	2.55E-09	2.67E+04	6.79E-05
RM20D	week 25	rh2011	n.d.	n.d.	n.d.
RM20E	week 53	rh2011	n.d.	n.d.	n.d.
RM20E1	week 53	rh2011	5.85E-10	5.75E+04	3.36E-05
RM20E2	week 53	rh2011	n.d.	n.d.	n.d.
RM20E3	week 53	rh2011	n.d.	n.d.	n.d.
RM20F	week 53	rh2011	1.60E-08	8.11E+03	1.30E-04
RM20G	week 53	rh2011	8.01E-11	5.71E+05	4.58E-05
RM20H	week 53	rh2011	1.61E-08	1.37E+04	2.21E-04
RM20I	week 53	rh2011	1.27E-07	2.53E+03	3.21E-04
RM20J	week 53	rh2011	<1.0E-12	4.47E+04	<1.0E-07
