# Supplementary material for: Mapping the immunogenic landscape of near-native HIV-1 envelope trimers in non-human primates
Source: PLoS Pathog. 2020 Aug 31;16(8):e1008753. doi: 10.1371/journal.ppat.1008753 (PMC7485981; doi:10.1371/journal.ppat.1008753)
Supplement: S7 Table — (PDF) [file ppat.1008753.s011.pdf]

S7 Table. Heterologous neutralization RM20F.

Virus name	Tier*	Subtype	Accession	RM20F	ACS202*	VRC34*
001428_2_42	2	C	EF117266	>100	>10	0.075
1012_11_TC21_3257	1B or 2	B	EU289184	>100	>10	n.d.
AC10_29	2	B	AY835446	>100	>10	>10
BJOX010000_06_2	2	01_AE	HM215373	>100	0.506	>10
BJOX015000_11_5	2	01_AE	HM215377	>100	0.023	n.d.
BJOX025000_01_1	2	01_AE	HM215386	>100	0.003	>10
BJOX028000_10_3	2	01_AE	HM215389	>100	0.005	>10
C2101_C1	2	01_AE	JN944661	>100	n.d.	0.218
C3347_C11	2	01_AE	JX512902	>100	>10	>10
C4118_9	2	01_AE	JQ352782	>100	0.411	>10
CAP210_E8	2	C	DQ435683	>100	0.297	0.183
CAP45_G3	2	C	DQ435682	>100	n.d.	0.056
CNE20	2	07_BC	HM215406	>100	>10	>10
CNE5	2	01_AE	HM215415	>100	>10	>10
DU156_12	2	C	DQ411852	>100	n.d.	>10
DU172_17	2	C	DQ411853	>100	n.d.	0.078
PVO_4	2 or 3	B	AY835444	>100	0.043	>10
Q23_17	1B	A1	AF004885	>100	>10	0.099
Q842_D12	2	A1	AF407160	>100	n.d.	0.138
REJO4541_67	2	B	AY835449	>100	>10	>10
RHPA4259_7	2	B	AY835447	>100	0.012	1.559
SC422_8	2	B	AY835441	>100	>10	>10
THRO4156	2	B	AY835448	>100	n.d.	>10
TRJO4551_58	3	B	AY835450	>100	0.029	7.334
TRO_11	2	B	AY835445	>100	>10	>10
ZM109_4	1B or 2	C	AY424138	>100	n.d.	1.432
ZM135_10A	2	C	AY424079	>100	n.d.	>10
ZM197_7	1B or 2	C	DQ388515	>100	n.d.	>10

*Data obtained from Los Alamos National Lab (LANL) CATNAP application (Yoon et al., 2015).
