# Supplementary material for: Mapping the immunogenic landscape of near-native HIV-1 envelope trimers in non-human primates
Source: PLoS Pathog. 2020 Aug 31;16(8):e1008753. doi: 10.1371/journal.ppat.1008753 (PMC7485981; doi:10.1371/journal.ppat.1008753)
Supplement: Attachment — Submitted filename: response reviewers 20200617.docx [file ppat.1008753.s012.docx]

Original Reviewers comments in black, responses in red

**Part I - Summary**
Please use this section to discuss strengths/weaknesses of study, novelty/significance, general execution and scholarship.

Reviewer #1: In this manuscript, Cottrell et al. report the characterization of 42 new monoclonal antibodies isolated from two rhesus macaques previously immunized with the BG505 SOSIP.664 trimer. All of the antibodies bound the aforementioned trimer. Six of them neutralized the autologous pseudovirus BG505.T332N (with modest potency) and 9 neutralized a N611-glycan deficient variant BG505.T332.N611A (some with high potency). Out of the neutralizing antibodies, three neutralized both viruses. Extensive characterization of all 42 antibodies (neutralizing and non-neutralizing) revealed their antigenic specificities: 55% of the 42 antibodies bound the base of the trimer, 19% the 289-glycan hole, 12% an epitope near the N611 glycan, and 2% the gp120/gp41 interphase. The antibody with the most potent neutralizing activity against BG505.T332N bound the gp120/gp41 interphase in an epitope that overlaps with VRC34.01, a previously identified broadly neutralizing antibody of human origin.

In my opinion the data is clear, and the manuscript is well written and balanced. Remarkably, the authors did a big effort in putting together a comprehensive dataset of rhesus germline sequences to be used with their analyses. In fact, having a good database of sequences is important for proper identification of the corresponding germline genes and alleles, and this translates into accurate SHM rate calculations. Current databases are indeed limited, at least in regard to rhesus sequences. Importantly, the authors generated an online tool for antibody sequence analysis with their newly generated database: <http://ward.scripps.edu/gld/>. The tool/site was made freely available and will certainly be useful for such analyses. Hopefully more sequences will be added over time to keep it growing.

The antibody epitope analyses were very thorough, and I agree with the authors that extending those to all the antibodies rather than focusing only on the neutralizing ones was a good idea, with the ultimate goal of improving future designs of SOSIP trimers.

Reviewer #2: The manuscript by Cottrell et al. describes a series of in depth analyses of monoclonal antibodies generated from two rhesus macaques that were immunized using the HIV-1 Env BG505 SOSIP.664 trimer. These two macaques were chosen because they showed higher levels of neutralizing antibody after immunization. Similar experiments have been done in rabbits, and will soon be done with samples from humans being immunized with this immunogen. A comparative study is of value in assessing the predictive value of model systems given the small number of immunogens that will ever be tested in humans. The study is comprehensive in that it goes from the immunized animals all the way to the structure of mAbs bound to the trimer. This represents a serious attempt to characterize the nature of the antibodies raised in macaques by the SOSIP trimer. Even if it ultimately does not inform the nature of the immune response in humans it still represents an important data point on the path to vaccine development. In addition, the authors expand the number of rhesus macaque germline BCR alleles to help interpret somatic hypermutations. I have only a few minor comments.

Reviewer #3: In “Mapping the immunogenic landscape of near-native HIV-1 envelope trimers in non-human primates,” Cottrell et al. describe the isolation of 42 monoclonal antibodies from two Indian origin macaques, immunized repeatedly over a year with the BG505 SOSIP.664 immunogen. Sixteen of these antibodies were characterized for recognition by negative stain EM, to provide a general notion of epitope – then further characterized at higher resolution for three of the antibodies: RM20J, which recognized a glycan hole at residue 289; RM20F, which recognized an interface epitope; and RM20E1, which recognized a quaternary epitope including the fusion peptide. While only 6 of the 42 antibodies were capable of neutralizing the autologous BG505 virus, the authors argue – I believe correctly – that collectively, these antibodies delineate many of the most immunogenic features of the SOSIP-based immunogen. These antibodies and their mapping thus represent a helpful resource, delineating the dominant immunogenic characteristics of an important HIV-1 immunogen that has entered clinical trials.

We thank the reviewers for the positive and accurate summaries of our manuscript.

**Part II – Major Issues: Key Experiments Required for Acceptance**
Please use this section to detail the key new experiments or modifications of existing experiments that should be absolutely required to validate study conclusions.

Reviewer #1: (No Response)

Reviewer #2: None

Reviewer #3: While okay to focus on strain-strain specific and non-neutralizing antibodies, to provide perspective for their results, the authors should discuss/compare their antibodies with the few broadly neutralizing antibodies, which have now been elicited from macaques with modified SOSIP trimers (e.g. Dubrovskaya et al. 2019, Immunity and Kong et al. 2019, Cell). The authors cite these papers when they state, “Native-like Env trimers are an important design platform for engineering immunogens for bNAb induction.” (Lines 274-275), but they provide little comparison/context. They should compare their interface antibody (RM20F) with the interface antibody (1C2 with 87% breadth) from the Dubrovskaya manuscript and their fusion peptide antibodies (including RM20E1) with the fusion peptide antibody (DFPH-a.01 with 59% breadth) from the Kong manuscript. What are key differences between the broadly neutralizing antibodies versus the strain-specific or non-neutralizing ones characterized in their study? (SHM? Angle of approach?) While fine to focus on the diverse landscape of immunodominant antibodies, it’s important to provide context as to where broadly neutralizing antibodies elicited in macaques lie within that landscape.

We thank the reviewer for the suggestion and agree it is of importance to understand why some antibodies exhibit potent and broad neutralization capacity and others not. As point of clarification, the 1C2 mAb reported in the Dubrovskaya et al. 2019 was elicited in rabbits not rhesus macaques. Moreover, the mechanism for neutralization for 1C2 seems to be a prying apart of gp41 that results in trimer degradation similar to the human bnAb 3BC315. 1C2 does not interact with the FP and was elicited using an immunogen in which the FP is covalently bound to the c-terminus of gp120 via a glycine/serine flexible linker. We saw no evidence of trimer degradation following incubation of the BG505 Env trimer with either RM20E1 or RM20F. 1C2 does overlap with the epitopes of RM20E1 and RM20F.

There is no structure of the DFPH-a.01 mAb bound to a trimer available. We compared our mAbs to a DFPH-a.01 lineage member (DFPH-a.15), which has less breadth but for which a structure was available. RM20E1 or RM20F both interact with the FP similar to the DFPH-a.15 mAb. RM20E1 lacks neutralization breadth because it cannot neutralize viruses (including WT BG505) with a glycan at position N611 as shown in figure 1. RM20F lacks neutralization breadth because of its reliance on BG505 specific residues, which is explained in lines 231 and 250. Figure 5D has been updated to include a comparison of 1C2.

**Part III – Minor Issues: Editorial and Data Presentation Modifications**
Please use this section for editorial suggestions as well as relatively minor modifications of existing data that would enhance clarity.

Reviewer #1: I only have some minor comments/questions:

- The neutralizing antibodies described here targeted varied epitopes. For instance, RM19M (which neutralized both BG505.T332N and BG505.T332N.N611A) bound the base of the SOSIP, but others bound the glycan hole, etc. For enhanced clarity, I suggest including a supplementary table in which the info from the tables in Fig 1C and D is added and combined (adding extra columns) with the ELISA reactivity to SOSIP and gp120, as well as the information on the corresponding epitope for each antibody.

We thank the reviewer for the excellent suggestion. We have added Table S6 that combines the neutralization data, ELISA binding data, and EM epitope mapping into one table.

- A total of 42 antibodies were isolated (and 23 out of those bound the base of the trimer). Would the 19 remaining antibodies be representative or enough to cover all of the non-neutralizing epitopes found on the SOSIP trimer?

That is an excellent question. Using electron microscopy polyclonal epitope mapping (EMPEM) with serum of these animals and BG505 trimer, we observed that similar epitopes were targeted in both animals and that the epitope assignments in the serum correlated well with the mAbs generated by antigen-specific B-cell sorting. However, immunization with the BG505 trimer most likely elicits other non-neutralizing antibodies that are not capable of binding to the BG505 trimer probe and were therefore not detected using EMPEM and antigen sorting. The epitopes for these additional non-neutralizing antibodies are exposed as the BG505 trimer is degraded *in vivo* and include the tip of the V3 loop and perhaps the surface of gp120 that is occluded by the adjacent protomers in the SOSIP trimer. V3-targeting antibody responses are elicited frequently by BG505 trimers as shown by serum binding to V3 peptides and neutralization of tier 1 viruses. The B cells recognizing these epitopes are frequently discarded in cell sorting strategies (trimer negative, gp120 positive), as was done in this study as well and therefore these mAbs were not isolated in this study.

- What was the criteria used to select the 40 viruses used to test neutralization of RM20F and VRC34 in Figure S4?

The viruses tested for heterologous neutralization were selected as they represent the global diversity of HIV-1 (deCamp *et al.* JVI 2014). This panel includes the well-known 12-virus global panel extended by additional viruses to increase virus diversity based on both genetic and neutralization sensitivity characteristics. This panel is used standardly by the reference laboratory at Duke University by Dr. Montefiori and this neutralization was also performed there. This enables us to compare the data with data from other studies.

- If authors were trying to obtain as many sequences as possible for their database, why were those that were not identical to the one reference genome eliminated? In line with this, how many different animals are represented in the germline sequence database?

The database we started with was from the Vigdorovich et al., 2016 study and contained a mixture of gene/alleles from Indian and Chinese origin RMs. We compared this database to the only Indian origin RM reference genome that was available at the time in an effort to eliminate gene/alleles from Chinese origin RMs. As additional Indian origin RM genomic data became available, we added those gene/alleles to our database. Overall, our germline database contains genomic data from 12 Indian origin RMs and RepSeq (inferred genes/alleles) data from an additional 27 Indian origin RMs. This has been clarified in the methods and results section.

Reviewer #2: 1. The authors pick out two immunized macaques to analyze form mAbs. While they state these were the best two (out of four?) for neutralizing titers it is hard for the reader to see the data that is the basis for this choice. Perhaps it is in their earlier paper from 2015, which seems to be about immunized rabbits for the most part. Including a figure or table showing the neutralization titer and Tier 2 breadth of the starting serum from either these two or even all of the animals that were in the study where these two were included would be useful. This is important because there will be multiple studies like this and it will be informative to understand as much about the animal immune response to allow comparison between studies.

We are sorry for the confusion. Animals rh2011 and rh1987 were the only animals in a group of 4 RMs that were immunized with the BG505 SOSIP that developed autologous neutralizing serum activity (reported in Sanders et al., 2015). The titers ranged from ~1:125 to ~1:300 over time and no Tier 2 breadth was observed. We have now included the neutralization titers in line 117.

2. It would be useful to include in the Discussion a comparison of antibodies in rabbits, macaques, and humans (and maybe guinea pigs). As one example, who can make long CDR3 loops and how often is this an important feature of a neutralizing antibody? More generally, what are the important features of neutralizing antibodies and how well do these different species generate antibodies with the relevant features? This would help the reader frame how to compare results in different species, ultimately to help understand which will be most informative in modeling humans.

We thank the reviewer for the suggestion. We provided a comparison of RM20J with the BG505 SOSIP elicited rabbit mAb 10A that targets an overlapping epitope (Fig 3D and 3E) and in lines 209-213. Additional comparisons between rabbit and RM antibodies are complex and are beyond the scope of this paper. No human mAbs elicited with the BG505 SOSIP are available yet and the guinea pigs mAbs elicited using the BG505 SOSIP do not overlap with the rabbit or RM mAbs.

Reviewer #3: In terms of minor issues with presentation: the figures are generally clear, and well labeled, though yellow fonts should be darkened, and some of the writing should be improved.

We agree with the reviewer and have darkened the yellow fonts in the figures.

The first two sections of the results should be edited to move methodological (what was done) details to Methods; this first part of the results paper makes the case for accurate SHM determination, though I don’t see SHM information highlighted in the description of structures – only in the penultimate paragraph of the paper.

We thank the reviewer for the suggestion. We have edited the first two sections of the Results to remove methodological descriptions, which have been moved to the Methods section.

Information on lines 123-147 seems better suited to a table.

We thank the reviewer for the comment and agree that these lines are very dense with data. We have reported the summary of the information in Fig 1B and in greater detail in Table S2 if readers want to dive into the details. In the text we provide an overview of the mAb characteristics and highlight some clonal families.
